# Efficient Knock-in by CRISPR/Cas9-mediated homology-directed repair in *Caenorhabditis briggsae*

**DOI:** 10.17912/micropub.biology.001203

**Published:** 2024-07-10

**Authors:** Jiaonv Ma, Wangyan Zhou, Wenhua Shao, Yongbin Li

**Affiliations:** 1 College of Life Science, Capital Normal University, Beijing, Beijing, China

## Abstract

The CRISPR/Cas9 technology opens new avenues for detailed genetic exploration and comparative genomics. However, the current application of CRISPR/Cas9 in *C. briggsae* especially for homology-directed repair (HDR) knock-in (KI) still encounter significant challenges. In this study, we demonstrate that employing high concentration HDR donor vectors significantly improves KI efficiency in *C. briggsae* .

**Figure 1. Cas9-Induced HDR and Transgene Expression in C. briggsae:**
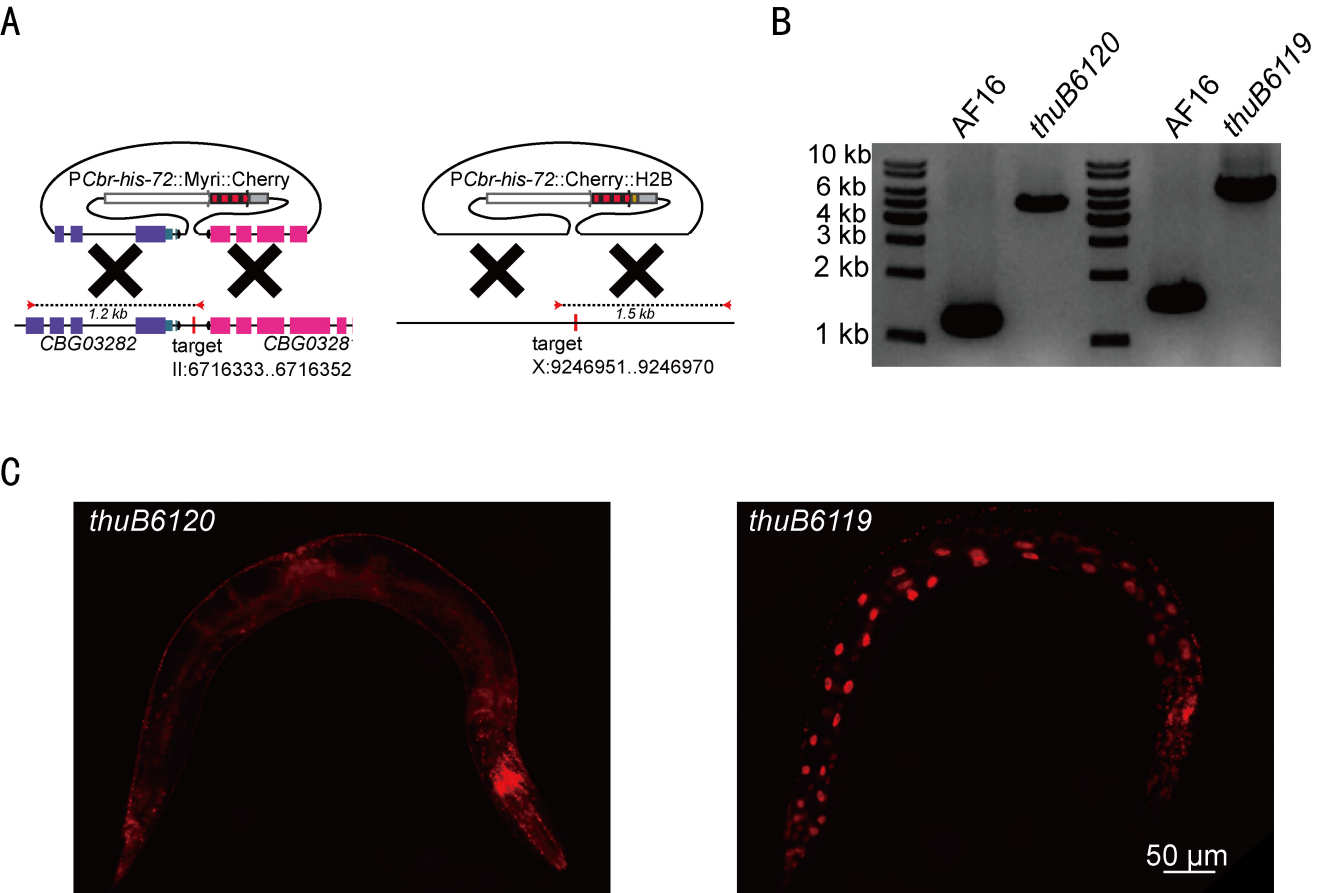
**(A)** Schematic diagrams of vector constructs for targeted homologous recombination. The vector P *Cbr-his-72* ::Myri::Cherry (left) targets a site flanked genes *CBG03282* and *CBG03281* at positions II: 6716333..6716352. The vector P *Cbr-his-72* ::Cherry::H2B (right) is designed for targeting a site at position X:9246951..9246970. **(B)** PCR genotyping of the indicated alleles using primer pairs as schematized in (A). **(C)** Fluorescent microscopy demonstrates transgene expression in *C. briggsae* . The image on the left ( *thuB6120* ) displays expression of P *Cbr-his-72* ::Myri::Cherry indicative of targeting chromosome II. The image on the right ( *thuB6119* ) shows expression of P *Cbr-his-72* ::Cherry::H2B consistent with targeting of chromosome X. Scale bar: 50 µm.

## Description

The advent of CRISPR/Cas9 technology has significantly advanced genome engineering, enabling precise genetic sequence editing across a variety of organisms. *Caenorhabditis briggsae* , closely related to *C. elegans* , is becoming increasingly popular for comparative biological studies due to its genetic similarities and distinct evolutionary trajectory (Gupta et al., 2007) . However, the application of CRISPR/Cas9 in *C. briggsae* has still faced challenges, especially for HDR-based long fragment KIs (Culp et al., 2020) , which are essential for comparative and functional genomics studies. While conventional protocols using the P *eft-3* promoter-based CRISPR/Cas9 KI in *C. elegans* often do not require high concentrations HDR-donor vectors (Dickinson et al., 2013), this approach may not be suitable for protein-based CRISPR/Cas9 protocols in *C. briggsae* (Culp et al., 2020) .

To accomplish Cas9-induced HDR in *C. briggsae* , we designed two sgRNAs targeting specific loci on chromosomes II and X in *C. briggsae* , validated through in vitro cleavage assays for maximum sgRNA efficiency. We then created two homologous repair templates with approximately 1 kb homology arms for P *Cbr-his-72* ::Myri::cherry on chromosome II，which encodes the membrane-targeted myristoylated Cherry reporter,and P *Cbr-his-72* ::cherry::H2B on chromosome X ( Figure 1A ).

Initially, we injected AF16 with CRISPR-Cas9 ribonucleoproteins and homologous repair templates at concentrations similar to those typically utilized in *C. elegans* for vector-based Cas9 or Mos1 KIs (50~100 ng/µL final for repair templates), alongside a *Cbr-dpy-10* co-CRISPR sgRNA (Cohen & Sternberg, 2019) . We injected each donor vector at 50 ng/µL into 80 P0s. We achieved KIs in 4/442 (0.9%) and 0/371 (0%) of the screened F1 dumpy worms by PCR genotyping for the chromosome II and X loci, respectively. Given the verified efficiency of the sgRNAs through in vitro cleavage, it is unlikely that the low success rate of KI was due to sgRNA efficiency. Therefore, we redirected our attention to the concentration of the donor vector. We increased the concentration of the donor vector to 484 ng/µL for the chromosome II target donor vector and 445 ng/µL for the chromosome X target donor vector. By injecting 80 P0s again for both donor vectors, we achieved KIs in 18/400 (4.5%) and 1/152 (0.65%) of the F1 dumpy worms screened by PCR genotyping, respectively. These efficiencies are comparable to that of vector-based CRISPR/Cas9 KIs in *C. elegans* . These results suggest that increasing the donor vector concentration significantly enhances KI efficiency in *C. briggsae* .

For each locus, we maintained one strain with allele names *thuB6120* (chromosome II) and *thuB6119* (chromosome X) ( Figure 1B ). These alleles were subsequently backcrossed with *Cbr-she-1* ( *v49* ) to eliminate the dumpy mutation. Both alleles exhibited ubiquitous expression patterns in somatic cells ( Figure 1C ), indicating that these loci could serve as potential sites for somatic expression with single-copy insertion transgenic operations, similar to MosSCI, using Cas9. Although both loci exhibit germline silencing, this issue could potentially be addressed by employing PATCs introns (Aljohani et al., 2020) . Moreover, it is necessary to test more loci to fully evaluate their suitability and efficiency for transgenic applications.

While the chromosome II locus showed higher KI efficiency, using it for single-copy insertion transgenic operations poses a complication due to its proximity to the *Cbr-dpy-10* co-CRISPR site. The *Cbr-dpy-10* locus is located at approximately 5.28 Mb, while the chromosome II locus is at around 6.72 Mb, with only a 1.44 Mb distance between them. Currently, it is not feasible to directly correlate physical distance with genetic distance. Based on observations in *C. elegans* , gene-dense regions near the center of autosomes have significantly reduced rates of recombination, with the lowest rate being approximately 0.5 cM/Mb (Barnes et al., 1995, p. 141). Although there are no detailed reports on recombination rates in *C. briggsae* , it is plausible that *C. briggsae* exhibits similar patterns. Given that the entire chromosome II of *C. briggsae* is around 16 Mb long and that 6.72 Mb is close to the center, we can reasonably hypothesize that 1.44 Mb corresponds to approximately 1.44 cM. This means that for every 100 worms, about 1.44 would be recombinant. In practice, even after typically backcrossing five times, it is still necessary to screen hundreds of worms to obtain transgenic individuals without the dumpy phenotype.

In the future, when a genome editing event occurs at chromosome II locus close to and more left than that of *Cbr-dpy-10* co-marker, our *thuB6120* allele can be used as a balancer to reduce the effort of removing the linked *Cbr-dpy-10* mutation. Crossing the *thuB6120* homozygous strain with the strain containing the new transgene and its tightly linked *Cbr-dpy-10* mutation will produce F1 progeny heterozygous for all three alleles. In the F2 generation, individuals that are homozygous for the new transgene are likely homozygous for *Cbr-dpy-10* and do not carry the *thuB6120* allele, exhibiting the dumpy phenotype and no *Cbr-his-72* reporter fluorescence. On the other hand, recombinants that are homozygous for the new transgene with no *Cbr-his-72* reporter expression and do not exhibit the dumpy phenotype can be identified using fluorescence microscopy. This method significantly reduces the screening effort.

We did not systematically evaluate the effect of different plasmid concentrations on KI efficiency. However, the observed differences between low- and high-concentration donor vectors in protein-based Cas9 systems highlight the complexities of genome editing in nematode. A potential explanation is that, in vector-based Cas9 systems, the donor and Cas9 expression vector first enter the nucleus and form an extrachromosomal array, with protein expression occurring after plasmid DNA nuclear import. Consequently, when Cas9 ribonucleoproteins cleave DNA, high-copy arrays of donor DNA are already present in the nucleus, facilitating the contact between the cut site and the donor vector. Conversely, in protein-based Cas9 systems, the donor vector may enter the nucleus less efficiently than the Cas9 ribonucleoproteins. If the donor vector concentration is too low in this context, the probability of the cut site encountering the donor vector is small, increasing the likelihood of repair via the non-homologous end joining (NHEJ) pathway over homologous recombination. In another word, low concentration of donor vector decreases the frequency of HDR-based KI.

In summary, our findings provide compelling evidence for the successful application of CRISPR/Cas9-mediated genome editing in *C. briggsae* , particularly with high concentrations of HDR donor vectors. Our findings contribute valuable insights into the optimization of CRISPR/Cas9 protocols for nematodes, enhancing the toolkit for comparative and functional genomics research in *C. briggsae* .

## Methods

*C. briggsae* worms were cultured at 25ºC on NGM plates supplemented with OP50 E. coli .

The high concentration of vectors was obtained by combining multiple plasmid minipreps and then purifying and concentrating vectors through VAHTS DNA Clean Beads. Cas9 Ribonucleoprotein mixtures were prepared similar as described in (Paix et al., 2015) . The 1.6 µL Cas9 protein (15 mg/mL), 1.4 µL target sgRNA (150 µM), 0.4 µL *Cbr-dpy-10* co-CRISPR sgRNA (150 µM), 1.3 µL dsDNA donor vector and 0.3 µL 4M KCl were mixed together. The mixture was incubated at 37 °C for 15 minutes and then centrifuged at 13000 rpm at 4°C for 10 min before injection.

PCR genotyping of F1 dumpy worms was performed to screen for successful KI animals.

## Reagents

In this study, all sgRNAs were synthesized by GenScript Biotech as 1.5 nmol powder. Each sgRNA was prepared as 150 µM stock solution with water. The sgRNA sequences used in this study were *Cbr-dpy-10* co-CRISPR sgRNA (5'- ATTCGCGTCAGATGATGTAC-3'), ChrII sgRNA (5'-GCTCTATAAAGGCACCGCGG-3'), and ChrX sgRNA (5'-CGTGGGGAAAGTCTGCGCCC-3'). Genotyping of the KI regions was conducted using specific primers: for chromosome II, 5'-TGTGGAGAATGTTGGGTTCA-3' (forward) and 5'-ACACCTTCGACCCTCCTTTT-3' (reverse); for the X chromosome, 5'-ATTTTCGTTTGATTTGGTCAAAAACCA-3' (forward) and 5'-CAGCTGTCCCTCTCATTCCAAA-3' (reverse).

Recombinant *S. pyogenes* Cas9 Nuclease [homemade as descried in (Paix et al., 2015) , stock solution 15 mg/mL ].

VAHTS DNA Clean Beads (Vazyme Biotech, N411-01)

**Table:** 

Strain	Genotype	Available from
AF16	*C. briggsae* wildtype	CGC
RE770	*Cbr-she-1* ( *v49* ),IV	Guo Y, Lang S & Ellis RE, 2009
XIL6119	*thuB6119* (P *Cbr-his-72* ::Cherry::H2B:: *Cbr-his-72* 3'UTR),X	Xiao Liu Lab
XIL6120	*thuB6120* (P *Cbr-his-72* ::Myri::Cherry:: *Cbr-his-72* 3'UTR),II	Xiao Liu Lab
